# Delivery of Orally Administered Digestible Antibodies Using Nanoparticles

**DOI:** 10.3390/ijms22073349

**Published:** 2021-03-25

**Authors:** Toshihiko Tashima

**Affiliations:** Tashima Laboratories of Arts and Sciences, 1239-5 Toriyama-cho, Kohoku-ku, Yokohama, Kanagawa 222-0035, Japan; tashima_lab@yahoo.co.jp

**Keywords:** orally administered monoclonal antibodies, mouth-to-systemic circulation monoclonal antibody delivery, drug delivery system, neonatal Fc receptor-mediated transcytosis, oral immunotherapy, nanodelivery

## Abstract

Oral administration of medications is highly preferred in healthcare owing to its simplicity and convenience; however, problems of drug membrane permeability can arise with any administration method in drug discovery and development. In particular, commonly used monoclonal antibody (mAb) drugs are directly injected through intravenous or subcutaneous routes across physical barriers such as the cell membrane, including the epithelium and endothelium. However, intravenous administration has disadvantages such as pain, discomfort, and stress. Oral administration is an ideal route for mAbs. Nonetheless, proteolysis and denaturation, in addition to membrane impermeability, pose serious challenges in delivering peroral mAbs to the systemic circulation, biologically, through enzymatic and acidic blocks and, physically, through the small intestinal epithelium barrier. A number of clinical trials have been performed using oral mAbs for the local treatment of gastrointestinal diseases, some of which have adopted capsules or tablets as formulations. Surprisingly, no oral mAbs have been approved clinically. An enteric nanodelivery system can protect cargos from proteolysis and denaturation. Moreover, mAb cargos released in the small intestine may be delivered to the systemic circulation across the intestinal epithelium through receptor-mediated transcytosis. Oral Abs in milk are transported by neonatal Fc receptors to the systemic circulation in neonates. Thus, well-designed approaches can establish oral mAb delivery. In this review, I will introduce the implementation and possibility of delivering orally administered mAbs with or without nanoparticles not only to the local gastrointestinal tract but also to the systemic circulation.

## 1. Introduction

Medicines benefit human health. Oral administration of medications is the most convenient and commonly used route from the standpoint of quality of life. However, barriers due to insolubility in water, gastric juice with low acidity, degradative or metabolic enzymes, and the epithelial cell membrane impede drug delivery. In particular, peptides, proteins, and nucleic acid drugs are associated with almost all these factors. In contrast, low-molecular-weight drugs are pharmacokinetically appropriate for oral administration because of their enzymatic stability and membrane permeability, which result in good absorption, distribution, metabolism, and excretion (ADME). They enter the cells across the membrane not only through passive diffusion but also through carrier-mediated transport by solute carrier transporters. However, at present, drug modalities have become diverse owing to the depletion tendency of drug seeds or drug discovery targets. Generally, compounds are categorized into three groups: low-molecular weight compounds (molecular weight (MW) < approximately 500), high-molecular weight compounds (MW > approximately 3000), and medium-molecular weight compounds (MW approximately 500–3000). While low-molecular weight drugs, as the mainstream pharmaceutical agents, have achieved the best success among these groups, high-molecular weight compounds and medium-molecular weight compounds have further scope for development. Multiple intravenous monoclonal antibodies (mAbs) that are high-molecular weight compounds have been approved by the U.S. Food and Drug Administration (FDA); they are used in clinical practice for the treatment of various diseases. High-molecular weight compounds such as protein and nucleic acid drugs, as well as medium-molecular weight compounds such as peptide drugs, have great potential to satisfy unmet medical needs. Therefore, the oral administration of peptides or proteins with or without nanodelivery systems has been proactively explored in drug development [1,2,3]. However, the methodology for oral mAb delivery has not yet been established [4].

Drug delivery systems are highly important in solving barrier problems, such as transmembrane transportation in drug discovery and development. I have described a transporter-conscious drug design for crossing the cell membrane based on carrier-mediated transport [5], delivery of substances into cells across the membrane using cell-penetrating peptides through endocytosis or direct translocation [6], drug internalization into cancer cells that express specific proteins to avoid off-target side effects in cancer therapy [7], substance delivery into the brain across the blood–brain barrier (BBB) based on transcytosis [8], and intranasal conjugated substance delivery into the brain using insulin as a carrier [9]. Despite these approaches, problems in drug delivery persist. It is true that intravenous mAbs provide significant patient benefit; however, the patients are subject to the inconvenience of injection or drip infusion, compared to peroral administration. In this perspective review, I will introduce the implementation and possibility of the delivery of orally administered mAbs to the target sites.

## 2. Discussion

### 2.1. The Small Intestinal Epithelium

**Figure 1:**
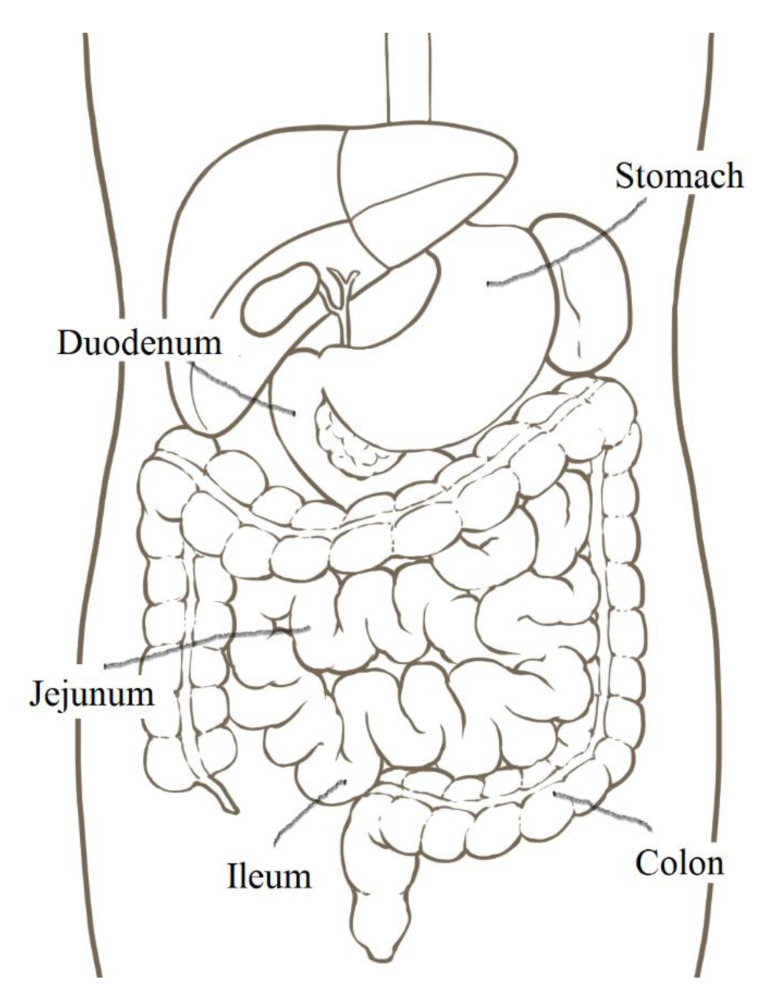
The position of digestive organs.

Oral administration of drugs is the most convenient and acceptable route because it is simple, economical, and safe. However, almost all mAbs approved by the FDA are intravenously or subcutaneously administered owing to enzymatic instability and difficulty in controlling their pharmacokinetics. When mAbs are administered orally, safety and simplicity are fulfilled. Thus, oral administration of mAbs poses important challenges. The trajectory of peroral drugs and foods based on peristalsis is described here (Figure 1). Drugs pass from the mouth to the stomach (approximately 30 cm in length, pH 1–3.5) through the esophagus (approximately 25 cm), and they are transported to the small intestine (approximately 7 m, pH 6.3–7.5), which is composed of the duodenum (approximately 25 cm), jejunum, and ileum. The jejunum forms the upper 2/5 parts of the jejunoileum, and the ileum forms the lower 3/5 parts of the jejunoileum, without a distinct boundary. The lamina propria in the jejunum is more vascularized than the lamina propria in the ileum. The intestinal villi in the small intestine increase the surface area for absorbing materials effectively. Most orally administered drugs are absorbed in the small intestine across the intestinal epithelium to the portal vein, leading to systemic circulation via enzymatic metabolism in the liver. The remaining drugs are conveyed to the colon (approximately 1.6 m, pH 7.5–8) and eventually expelled into the feces [10]. The mechanisms of substance absorption in the intestinal epithelial cells are classified as the transcellular pathway and paracellular pathway. Furthermore, the transcellular pathway is categorized into passive lipoidal diffusion, carrier-mediated transport based on solute carrier transporters, receptor-mediated transcytosis (RMT), and bystander transportation through pinocytosis. The paracellular pathway involves the passive diffusion of small solute materials such as ions and water through tight junctions between cells. mAb molecules are so large that they cannot pass through tight junctions, internal cavities of transporters, and cell membranes on the basis of passive lipoidal diffusion. Basically, mAbs are transported across the membrane through receptor-mediated endocytosis and spontaneous pinocytosis. Therefore, molecular design for achieving the delivery of oral mAbs or nanoparticles delivery should consider these facts.

**Figure 2:**
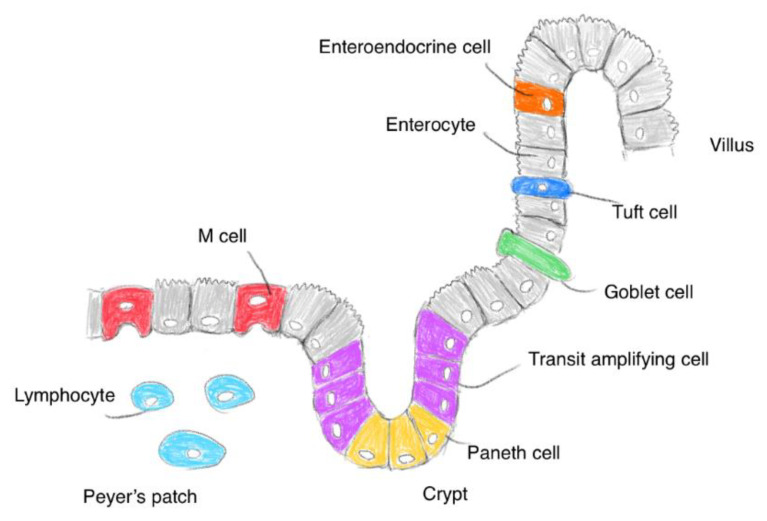
Pattern diagram of the small intestinal surface.

The small intestinal epithelium is primarily composed of enterocytes, microfold (M) cells, goblet cells, tuft cells, enteroendocrine cells, and Paneth cells (Figure 2). First, enterocytes, also called intestinal absorptive cells, play a vital role in the uptake of nutrients, vitamins, peptides, water, and ions. Most orally administered drugs are absorbed by enterocytes. Second, the gut-associated lymphoid tissue is composed of Peyer’s patches and isolated lymphoid follicles; it contains immune cells such as T and B lymphocytes, macrophages, antigen-presenting cells, and intraepithelial lymphocytes. M cells of Peyer’s patch transcytose antigens and present them to immune cells [11]. mAbs and nanoparticles are absorbed through endocytosis by M cells covered with a thin mucus layer. However, the population of M cells is considerably smaller than that of enterocytes. Third, goblet cells continually secrete mucin into the lumen. Mucins are negatively charged and composed of highly *O*-glycosylated glycoproteins; they form a single mucus layer (approximately 30 μm in depth) in the small intestine and two mucus layers in the colon [12]. The mucus layer averts abrasion on the epithelial surface by undigested food and the invasion of microorganisms and toxic materials. In addition to materials essential for life activity, drugs penetrate the mucus layer into the epithelial cells. Thus, orally administered mAbs or nanoparticles must penetrate the mucus layer in order to elicit their activity at target sites, except for the intestinal lumen. Fourth, tuft cells function as chemosensory sentinels to regulate type 2 immune responses to parasites and protozoa by producing biological effector molecules [13]. Fifth, various enteroendocrine cells secrete cell-specific endocrine hormones or hormone-like substances, such as somatostatin, glucagon, and serotonin. Sixth, Paneth cells in the crypts secrete antimicrobial peptides. The gastrointestinal tract, which connects to the external environment, possesses a highly developed system for immune defense against exogenous materials and microorganisms, in addition to digestive and absorptive systems. Eaten foods are digested by digestive enzymes such as pepsin in the stomach and trypsin, chymotrypsin, carboxypeptidase, and elastase in the small intestine. Peroral mAbs are exposed to these digestive enzymes. In fact, mAbs are denatured by pepsin at pH 2, whereas they are denatured to some extent by trypsin or chymotrypsin at pH 8. Drugs are considered to be pharmacokinetically influenced by structural and biological systems. Accordingly, drug design for oral administration should be conducted in consideration of anatomical and biological features in the gastrointestinal tract and stomach, particularly in the enterocytes and M cells of the small intestine.

### 2.2. Viruses Entering the Intestinal Epithelium

Human norovirus (HuNoV), a non-enveloped, single-stranded RNA virus, enters the body from the mouth through the gut tract and causes diarrheal illness. Its capsid (23–40 nm in diameter) protects RNAs from gastric acid and digestive proteases, as if it functioned as a nanoparticle carrier. VP1, a major capsid protein derived from HuNoVs, was detected in enterocytes, macrophages, T cells, and dendritic cells in intestinal biopsies. In particular, enterocytes were found to be relevant to HuNoV replication [14]. Moreover, enteroendocrine cells are relevant to HuNoV replication [15]. It has been proposed that the mechanism underlying the internalization of HuNoVs into cells is based on receptor-mediated endocytosis [16]. Therefore, it was suggested that unstable materials such as proteins and nucleic acids could be orally delivered using nanoparticles as carriers that protect them from gastric acid and digestive proteases. For rational molecular design and drug development, medicinal chemists and pharmaceutical scientists are utilizing evasion strategies used by viruses.

### 2.3. Rise of Intravenous Antibody Drugs on the Market

mAb drugs show high specificity to target substances, such as antigens, and are called molecular-targeted agents. Muromonab-CD3 (anti-CD3) was approved by the FDA as the first mAb in 1986. Since then, several mAb drugs have been approved [17]. Currently, pharmaceutical companies have produced revenue from the sale of mAb drugs. The total global sales of mAbs were approximately US$115.2 billion in 2018 and projected to reach US$300 billion by 2025 [17]. The top 10 drugs with the highest worldwide sales in 2019 were: (1) Humira (adalimumab), (2) Keytruda (pembrolizumab), (3) Revlimid (lenalidomide), (4) Opdivo (nivolumab), (5) Eylea (aflibercept), (6) Eliquis (apixaban), (7) Enbrel (etanercept), (8) Avastin (bevacizumab), (9) Stelara (ustekinumab), and (10) Rituxan (rituximab). This list includes several mAb drugs. Thus, mAb drugs have shown promising results in the pharmaceutical market. However, depletion of the targets for mAb drug development and restricted patterns of antigen-binding sites on mAbs are serious problems. All approved mAb drugs are administered via parenteral routes. Peroral administration may be a solution in terms of product life, in addition to simplifying drug administration.

### 2.4. pH-Dependent Dissolution of Nanoparticles and Subsequent Peptide Cargo Release

**Figure 3:**
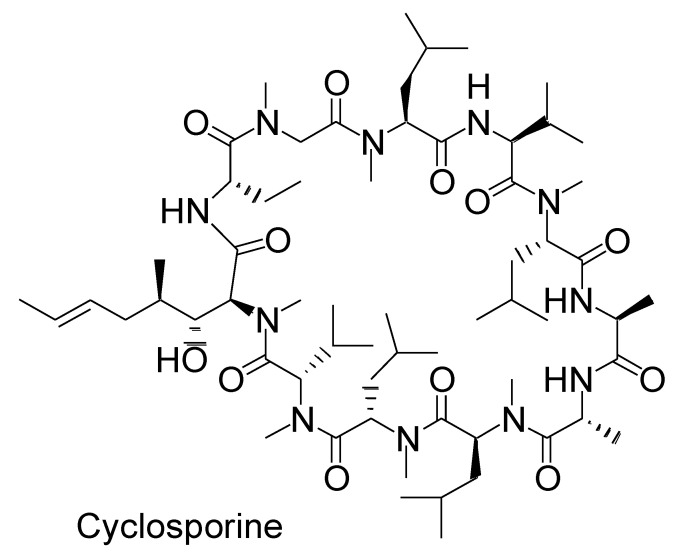
Structure of cyclosporine as *N*-methylated macropeptide.

Upon oral administration, macrocyclic peptides such as *N*-alkylated cyclic cyclosporin (1202.61 Da) (Figure 3) are absorbed through passive lipoidal diffusion without proteolysis in the small intestine, owing to their hydrophobicity, compact size, and enzymatic stability [18]. In general, peptides or proteins are digested in the stomach and small intestine. Thus, nanodelivery systems are required to protect orally delivered peptides or proteins from degradation. Fatty acid transport protein 4 (FATP4), a transporter that transports long-chain fatty acids (LCFA), with a carbon chain length of *n* > 12, was used for peptide delivery across the membrane using a nanodelivery system. The LCFA conjugate covalently linked with peptides such as exendin-4 (Ex4) (4186.57 Da), which is composed of 39 amino acids, was expected to cross the intestinal epithelium. The LCFA-Ex4 conjugate was released from LCFA-Ex4-loaded and chitosan-based nanoparticle-stabilized liposomes (OxaEx4) at pH 7, which is close to the pH in the small intestine, and was transported across the membrane according to an in vitro test using FATP4-positive Caco-2 cells as an epithelial cell model. This release was achieved through the sponge effect of chitosan. Furthermore, the long fatty acid chain protected the conjugated peptide from enzymatic degradation after its release from the carrier [19]. Various pH-sensitive nanoparticles have been developed [20]. Such well-conceived strategies can be adaptable to oral high-molecular mAb delivery; however, mAbs (IgG; approximately 150 kDa) cannot be transported by transporters.

### 2.5. Orally Administered mAbs for Localized Effect in Gut

**Figure 4:**
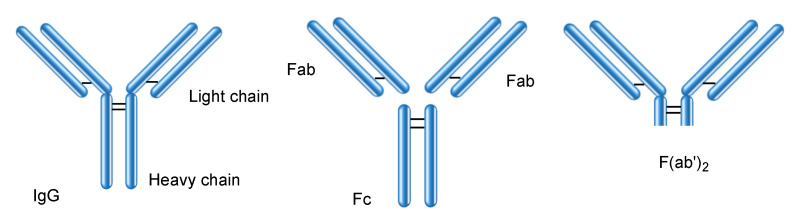
Structures of IgG and its fragments such as Fc, Fab, and F(ab′)_2_.

Immunoglobulins are categorized into five classes: IgG (Figure 4), IgA, IgM, IgD, and IgE. IgG antibodies are commonly used as mAb drugs. There are four IgG subclasses: IgG1, IgG2, IgG3, and IgG4. The IgG protein is approximately 14.2 nm in diameter [21]. The HuNoV capsid (23–40 nm in diameter) contains an IgG molecule inside. However, natural immunoglobulins such as milk immunoglobulins are bare and exposed to a severe environment in the living body. Peroral mAbs are believed to be denatured by gastric acid and proteases. It is true that pepsin at pH 2 denatures large mAbs through proteolysis; however, trypsin or chymotrypsin at pH 8 did not completely denature them. A small amount of intact mAbs survived through escaping degradation by gastric acid and digestive enzymes; however, Fc (approximately 50 kDa), Fab (approximately 50 kDa), F(ab′)_2_ (approximately 110 kDa) (Figure 4), and other small peptide fragments were cut out [10]. F(ab′)_2_ fragments were obtained through pepsin cleavage. Fab and F(ab′)_2_ fragments retained their neutralizing activity. Therefore, orally administered mAbs can neutralize their corresponding antigens, which are involved in local infection, local inflammation, and local neoplasms in the gastrointestinal tract. Several clinical trials have been performed for evaluating oral mAbs in the treatment of such localized diseases (Table 1).

Inflammatory bowel disease (IBD) is caused by excess tumor necrosis factor (TNF)-α in the lamina propria. V565 is an anti-TNF-α domain antibody developed for oral administration that resists intestinal proteases such as trypsin, chymotrypsin, and pancreatin. Orally administered V565 was detected in the lamina propria because of the colonic mucosal barrier loss caused by TNF-α and in the serum, according to an in vivo assay using colitis mice; however, V565 was not detected in the colonic mucosal tissue and serum of naïve mice [22]. Thus, V565 is effective against ulcerative colitis (UC) and Crohn’s disease, which are classified as IBD. The formulation of coated mini-tablets was engineered for clinical use to prevent V565 from being degraded by gastric acid and pepsin in the stomach and to release it in the lower small intestine. At present, a phase 2 clinical trial of oral V565 (NCT02976129) and a phase 1 clinical trial of oral V565 in capsules (NCT03705117) were conducted for the treatment of Crohn’s disease.

AVX-470, an anti-TNF antibody from the bovine colostrum, in delayed-release enteric-coated capsules that protect the contents from gastric acid were evaluated for UC treatment through oral administration. This phase 1 clinical trial showed an efficacy trend (NCT01759056) [23].

Anti-CD3 antibody elicited an immunological effect on UC. Intravenous muromonab-CD3, a mouse monoclonal antibody against CD3, was originally approved for the clinical treatment of acute rejection after organ transplantation in 1986. The biological mechanism of UC is the induction of regulatory T cells through anti-CD3 antibody uptake in gut-associated lymphoid tissue. Orally delivered muromonab-CD3 improved symptoms in the colon of patients with UC in a phase 2 trial using omeprazole, a proton pump inhibitor (NCT01287195) [24].

Nonalcoholic steatohepatitis (NASH) is a chronic progressive hepatic disorder caused by neutral lipid accumulation (steatosis), subsequent oxidative stress, elevated cytokine levels, and insulin resistance. Foralumab is the first fully human anti-CD3 antibody. Peroral anti-CD3 antibody binds to the CD3/T cell receptor complex on T cells in the gut wall and eventually induces regulatory T cells that suppress inflammation in the liver by modulating cytokines [25]. A phase 2 clinical trial was conducted in NASH and type 2 diabetes mellitus (T2DM) using oral foralumab. Omeprazole was co-administered to neutralize stomach acidity. However, this trial was withdrawn (NCT03291249) [25]. The trial results implied that a proton pump inhibitor was not sufficient to suppress acidity in the stomach. Accordingly, another tactic should be implemented to guard the mAbs.

In addition, phase 2 clinical trials were conducted for NASH using oral muromonab-CD3 with omeprazole (NCT01205087) [26] and for non-responder genotype-I chronic hepatitis C using oral anti-CD3 mAb with or without omeprazole (NCT01459419). Detailed accounts of these observations are not known.

Food allergies are abnormal immune responses to specific foods, including eggs, milk, and wheat, and are extremely common in children and adults. This immediate allergic reaction is caused by IgE against food antigens. In the most serious cases, it causes life-threatening anaphylaxis. The number of patients with food allergies is increasing. Accidents due to food allergies should be prevented. Omalizumab, a humanized mouse anti-IgE antibody, was studied for milk allergy in a phase 2 trial. It did not demonstrate efficacy with respect to desensitization and sustained unresponsiveness compared to the placebo (NCT01157117) [27]. Moreover, a phase 2 trial for peanut allergy was performed for ascertaining whether impairment of allergen-specific regulatory T cell function caused by food allergy was restored through oral omalizumab administration. According to the results, desensitization was likely acquired by the loss of peanut-reactive regulatory T cells expressing a Th2 cell-like phenotype (NCT01290913) [28]. A phase 3 clinical trial using omalizumab administered by subcutaneous injection is being planned and is recruiting patients with food allergies (NCT03881696). Although, as part of oral immunotherapy, anti-IgE antibody was orally administered in these studies, noticeable efficiency might not be observed, probably because of denaturation in the stomach. As the symptoms of food allergy are displayed throughout the body, such as the skin, gastrointestinal tract, respiratory system, and cardiovascular system, antigens in food are suggested to move in the body through the systemic circulation after intestinal absorption. Children dislike intravenous injections because of pain. Thus, oral anti-IgE antibodies have to be taken in the small intestine and be subsequently transported across the intestinal epithelium to the systemic circulation without losing their neutralizing activity.

Celiac disease (CD) is a chronic autoimmune disorder of the small intestine caused by the presence of gluten in grains as an antigen. Orally administered capsules of anti-gluten egg yolk antibodies, such as IgY (AGY), were evaluated in phase 1 (NCT01765647) and 2 (NCT03707730) clinical trials for CD.

Although the etiology of autism is unknown, it has been suggested that gastrointestinal immunopathology is related to autism. Oral Oralgam, a human immunoglobulin, improved gastrointestinal dysfunction in children with autism in a phase 2 clinical trial (NCT00110708) [29].

Toll-like receptor 3 (TLR3) is involved in the pathogenesis of viral infections, including influenza, and is located in the endosomal membrane as the sentinel against viral infection to induce interferons. Nonetheless, an antibody against the TLR3 FYW peptide exhibited efficiency in a mouse model of influenza [30]. TLR3 antagonization inhibits production of pro-inflammatory cytokines [34]. A phase 2 trial was conducted for common cold using oral homeopathic antibody as a tablet formulation against the TLR3 FYW peptide (TAO1); it showed efficacy against moderate-to-severe upper respiratory tract infection (NCT01651715) [30]. The tested tablets were dissolved in the mouth and were not swallowed.

Tablets of anti-influenza antibodies in the bovine colostrum were orally administered to verify their safety and efficacy against influenza in phase 1 clinical trials (NCT01026350).

Enterotoxigenic *Escherichia coli* (ETEC) expressed colonization factor 17 (CS17), whose fimbriae are composed of a polymerized major subunit (CsbA) forming its stalk and a minor tip subunit (CsbD). Orally administered anti-CS17 bovine milk IgG and anti-CsbD bovine milk IgG were evaluated for diarrhea caused by ETEC in phase 2 clinical trials. Anti-CS17 Abs demonstrated protection against CS17-expressing ETEC (NCT00524004) [31]. Moreover, ETEC expressed colonization factor I (CFA/I) fimbriae, which have a major pilin subunit (CfaB) and minor pilin subunit (CfaE). Anti-CFA/I bovine IgG and anti-CfaE bovine IgG were evaluated in a phase 1 clinical trial for diarrhea. Anti-CfaE Abs demonstrated protection against CfaE-expressing ETEC (NCT00435526) [32].

In a phase 2 trial, orally administered serum-derived bovine immunoglobulin/protein isolate (SBI) was evaluated as a dietary supplement for mucositis (NCT04239261). Oral SBI reduced the levels of intestinal fatty acid binding protein (I-FABP) and zonulin, which suggested an improvement in gut damage induced by chronic human immunodeficiency virus (HIV) infection (NCT01828593) [33]. Moreover, an oral SBI phase 2 trial was performed to investigate postoperative recovery of female reproductive cancer after surgery (NCT01867606). Oral SBI was evaluated to determine whether it improved the nutritional status of patients with advanced chronic obstructive pulmonary disease (COPD) with cachexia (NCT02067377), diarrhea-predominant irritable bowel syndrome (NCT02163213), and irritable bowel syndrome (NCT02609529). Thus, these findings imply that SBI is biologically effective against certain diseases.

Although few phase 3 trials have been performed using orally administered mAbs, several phase 2 trials have been conducted. Phase 2/3 trials of oral food additive colostrum-derived antibodies against *Clostridium difficile* for the treatment of *C. difficile*-associated diarrhea were withdrawn (NCT00747071). In fact, orally administered mAbs have not been used in clinical practice [4]. The disappointing results of these trials might have been because the antibodies were exposed to gastric acid and pepsin at pH 2 instead of intestinal proteases, resulting in denaturation. Thus, nanoparticles should be employed to avoid denaturation, similar to the utilization of capsids by HuNoVs. A formulation for intestinal protease-resistant V565 has already been developed using coated mini-tablets with an enteric Eudragit^®^ L100 polymer soluble at pH 6.0. Moreover, an oral formulation of foralumab has been developed by encapsulating it in enteric-coated capsules coated with substances such as Eudragit^®^ L30D/L100–55 to shelter from gastric acid and pepsin and to release the cargos at pH > 4–5 [35]; a phase 2 trial of this formulation is planned in progressive multiple sclerosis or Crohn’s disease.

### 2.6. Proteins Interacting with IgG

Intriguingly, a very small amount of orally administered intact mAbs can reach the systemic circulation without being digested by gastric acid and proteases [10]. Phenomenalistically, antibody drugs can be administered not only through the intravenous route but also the oral route. The proteins described below help mAbs cross the epithelium to the systemic circulation.

#### 2.6.1. FcRn for Salvation and Transcytosis

**Figure 5:**
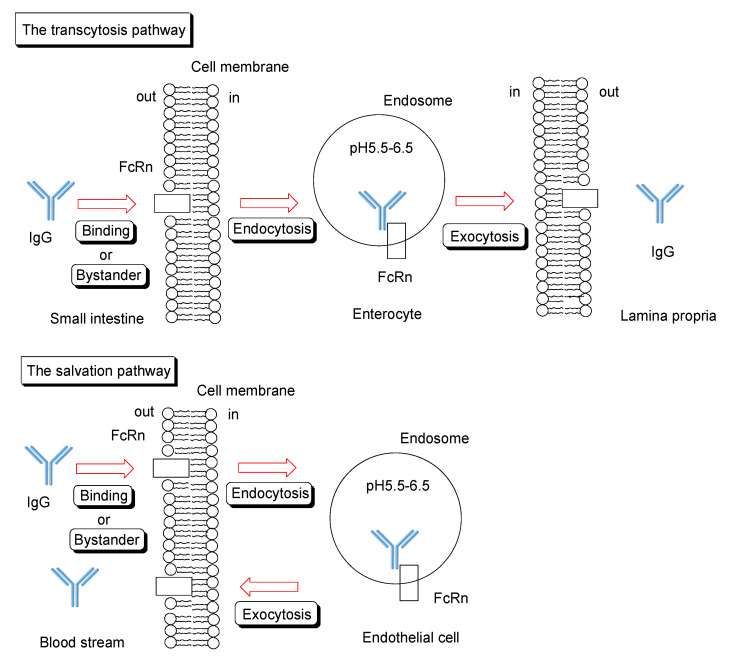
Schematic overview of transcytosis or salvation pathway of IgG through FcRn binding.

Neonatal Fc receptor (FcRn) [36,37] was recognized as the factor responsible for the absorption of maternal IgG antibodies in milk in the small intestine of babies. However, it was revealed that FcRn is expressed not only in babies but also in adults and possesses multifunctional roles in transporting, saving, and recycling certain materials such as IgG and albumin in the living body. FcRn is expressed in enterocytes of the small intestine, podocytes, renal proximal tubular cells of the kidney, syncytiotrophoblasts of the placenta, hepatocytes of the liver, and vascular endothelial cells [36]. FcRn binds to IgG under weakly acidic conditions (pH < 6.5), while FcRn does not bind to IgG under extracellular physiological pH between 7.0–7.4. Endosomes mature from early endosomes (approximate pH 6.5) to late endosomes (approximate pH 5.5) and, subsequently, lysosomes (approximate pH 4.5) through the effect of vacuolar H^+^-ATPase proton pumps. Therefore, the FcRn-IgG complex is formed in the early endosomes. Sorting to convey the contents through the lysosomal degradative pathway or the secretory pathway is conducted through division of the endosome into two. Endosomes containing the FcRn-IgG complex are fused to the plasma membrane. Endosomes without the FcRn-IgG complex mature into lysosomes, leading to degradation of its contents. Interestingly, ligands with high affinity to the transferrin receptor are subject to the degradative pathway, whereas those with moderate affinity are subject to the secretory pathway. FcRn plays a role in the salvation or transcytosis of IgG (Figure 5). Cells with FcRn perform their own mission. IgG molecules have a long half-life in the blood circulation because of salvation by endothelial FcRn. Salvation is observed in the vascular endothelial cells and hepatocytes of the liver. Transcytosis phenomena are observed in the enterocytes of the small intestine, podocytes [38], renal proximal tubular cells of the kidney, and syncytiotrophoblasts of the placenta. While dimeric IgA is transported in only one direction from the basolateral side to the apical side by the polymeric Ig receptor (pIgR), IgG is transported in both directions by FcRn. Moreover, even IgG-antigen complexes are transported from the apical side by FcRn and presented to antigen-presenting cells, such as dendritic cells. Thus, FcRn may play an important role in the immune system. An active transportation system for IgG-antigen complexes, different from pinocytosis, might be present in the small intestinal epithelium. Antigen-presenting cells, such as monocytes, macrophages, and dendritic cells, also express FcRn. It was suggested that the nascent phagocytic cup was acidified to induce FcRn-IgG binding during phagocytosis to enable internalization of opsonized bacteria by monocytes [39]. A similar system might function on the surface of enterocytes of the intestinal epithelium owing to Na^+^/H^+^ exchangers at their apical membrane.

#### 2.6.2. Trafficking of IgG and Albumin by FcRn

Endocytosed IgG binds to FcRn in endosomes through acidification. The IgG-FcRn complex is recycled back to the apical side or endocytosed to the basolateral side through the fusion between the endosomes and plasma membrane. After sorting and endosome maturation, other proteins are degraded in the lysosomes. At physiological pH, IgG is liberated from the FcRn. Similarly, the albumin-FcRn complex in endosomes is recycled or endocytosed. While IgG and albumin do not bind tightly to FcRn under physiological pH conditions, they bind to FcRn under weakly acidic conditions. Human IgG1-human FcRn exhibited a Kd value of 760 ± 60 nM at pH 5.8 [40]. The Kd values for humanized IgG (motavizumab)-human FcRn were 2140 nM at pH 6.0 and > 10,000 nM at pH 7.4 [41]. Human albumin-human FcRn showed a Kd value of 633.0 ± 97.0 nM at pH 5.5 and no detectable binding at pH 7.0 [42]. A homodimeric IgG (150–170 kDa) has two equivalent binding sites, one for each Fc region, with the same affinity for FcRn (40 kDa), consistent with the crystal structure. When the second FcRn binds to another unoccupied binding site on the IgG bound to the first FcRn, both the allosteric effect and steric hindrance do not occur according to the kinetic analysis, while a negative allosteric effect on the interaction between IgG-FcRn and another FcRn has been suggested in other studies. The variable region of IgG does not interact with Fc binding to FcRn [40]. Moreover, IgG and albumin (65–70 kDa) can bind simultaneously and independently to their own different binding sites on FcRn in a non-allosteric manner and without steric hindrance [43]. Accordingly, theoretically, heterodimers such as IgG-FcRn and FcRn-albumin, hetero-trimers such as IgG-(FcRn)_2_ and IgG-FcRn-albumin, hetero-tetramers such as IgG-(FcRn-albumin)-FcRn, and hetero-pentamer complexes such as IgG-(FcRn-albumin)_2_ [37] can exist under acidic conditions. However, it is unknown whether the trafficking routes of IgG and albumin differ with respect to such complex formation. In an in vitro assay using FcRn knockout mouse podocytes, IgG accumulated owing to IgG transcytosis impairment, and albumin did not accumulate without albumin transcytosis impairment [38]. It needs to be determined whether IgG∙FcRn and IgG∙(FcRn)_2_ exhibit different trafficking routes.

#### 2.6.3. IgG Transepithelium through RMT Based on Translational Substance Exchange

**Figure 6:**
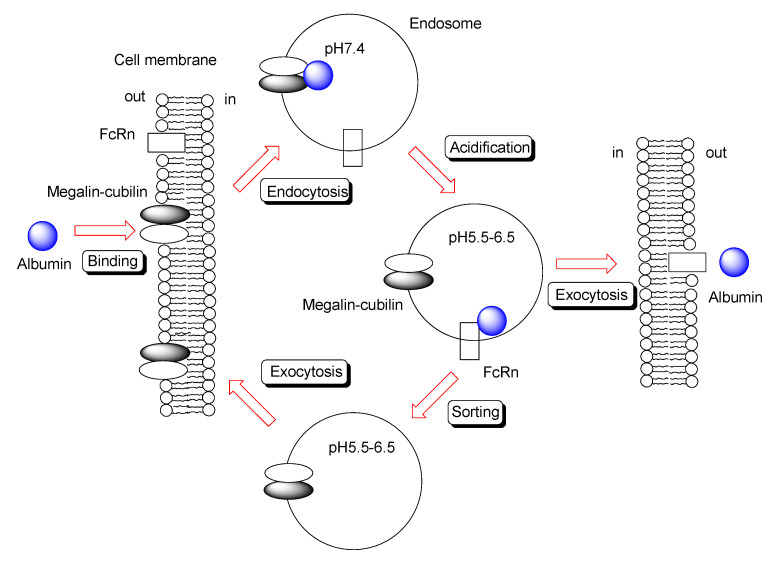
Schematic overview of transcytosis of albumin through megalin–cubilin and FcRn binding.

Under extracellular physiological pH, IgG antibodies do not bind tightly to FcRn on the apical membrane of endothelial and epithelial cells. Thus, they are believed to be internalized into cells through a fluid-phase endocytosis process, such as pinocytosis. However, fluid-phase endocytosis is not a ligand-specific process; the resultant transfer of bystander IgG into cells is not effective. Moreover, inherently, excess non-specific endocytosis is a disadvantage to the living body, because bystander harmful substances can enter cells to elicit toxicity. Therefore, receptor-mediated endocytosis using receptors other than FcRn is desirable for effective and specific internalization. Subsequently, IgG released from such an intervention receptor may bind to FcRn through acidification of the endosome, following which it can be released to the opposite extracellular space at physiological pH through the fusion between the endosome and basolateral membrane. Intriguingly, in renal tubular epithelial cells, albumin reabsorption occurs through this coordinated trafficking process via RMT. Albumin binding to the megalin-cubilin complex on the tubular lumen triggers clathlin-dependent endocytosis. Albumin transferred from the megalin-cubilin complex to FcRn in weakly acidified endosomes was released from FcRn to the peritubular capillary (Figure 6). Rat cubilin-rat albumin showed a Kd value of 630 nM at pH 7.0 [44]. The megalin-cubilin complex is expressed on the apical membrane of small intestinal epithelial cells in suckling rats [45]. FITC-labeled human IgG binds to the megalin-cubilin complex to induce clathrin-dependent endocytosis in opossum kidney epithelial cells [46]. Thus, IgG endocytosed in the small intestine, utilizing the megalin-cubilin complex as an intervention receptor, could be transferred to FcRn in endosomes through acidification (Figure 6). Megalin, cubilin, and FcRn were expressed in the small intestine [47]. Nonetheless, it has been suggested that megalin might not be expressed in the small intestine (Table 2) [48].

Furthermore, IgG is internalized through receptor-mediated endocytosis based on human FcγRI using INF-γ-treated U937 cells, where FcγRI expression is upregulated 4–5-fold, resulting in up to 60,000 receptors per cell. This IgG-FcγRI complex in endosomes is recycled back to the plasma membrane, while the IgG-FcγRI complex with cross-linking mAb against human IgG Fab is endocytosed and eventually degraded in lysosomes without being recycled [49]. Human FcγRI-human IgG1 has been shown to exhibit a Kd value of 2.9 nM [50]. Thus, IgG might be transcytosed in small intestine epithelial cells through RMT using megalin-cubilin or FcγRI as the first receptor complex and FcRn as the second receptor. Effective transepithelium of IgG in the small intestine can be accomplished using this strategy. However, although FcγRI is not expressed in the small intestine, cubilin, FcRn, and probably megalin are expressed (Table 2) [47,48]. Thus, a well-conceived molecular design is very important for achieving intended substance delivery to the target tissues and cells.

### 2.7. Possible Methods for Applying Orally Administered mAbs for Systemic Treatment across the Epithelium

Ideally, after internalization of nanoparticles containing mAbs through receptor-mediated endocytosis in the small intestine, mAbs should be released from acid-sensitive nanoparticles through acidification in endosomes, bound to FcRn, and exocytosed to the lamina propria. This strategy is suitable for intravenous nanoparticle delivery across the BBB. However, before they reach the small intestine, such acid-sensitive nanoparticles are activated to release their cargo in the stomach because of the gastric acid. Thus, nanoparticles sensitive to pH (6.3–7.5) in the small intestine, such as enteric Eudragit^®^, should be used for nanodelivery systems. Released mAbs could be transported across the epithelium to the lamina propria through four pathways: (1) RMT in enterocytes using megalin-cubilin as the first receptor, (2) RMT in enterocytes using FcRn as the first receptor, (3) phagocytosis, and (4) RMT in M cells. These transportations can occur simultaneously. Internalization through phagocytosis is considered a coincidental event. Therefore, nanoparticles with mAbs need to be designed for intelligent and effective internalization of released cargos through RMT. In seven neonates and infants who were exclusively breast-fed, the pH values were 6.4 ± 0.5 in the duodenum, 6.6 ± 0.4 in the jejunum, and 6.9 ± 0.7 in the ileum. Moreover, in eight neonates and infants who were fed solely cow’s milk, the pH values were 6.3 ± 0.9 in the duodenum, 6.0 ± 0.5 in the jejunum, and 6.3 ± 0.8 in the ileum [51]. These values were almost the same as those in adults. Nevertheless, peroral IgG antibodies were absorbed in the small intestine of neonates because of FcRn but not in adults. Milk feeding in neonates neutralizes the gastric pH to 4–5 [10]. Neonates have an immature digestive system. Thus, it is suggested that undigested intact IgG may be transported in the small intestine under such conditions. This can be reproduced using nanoparticles that protect IgG from enzymatic and acid digestion and release it into the small intestine. Eventually, the mechanism for transepithelium is assumed to be, ideally, FcRn-mediated transcytosis or, realistically, megalin-cubilin and FcRn-mediated transcytosis through handoff. However, strategies using pH-sensitive nanoparticles as carriers have not yet been implemented. The possibilities for their implementation are described in more detail below.

#### 2.7.1. Oral mAb Delivery Using Nanoparticle through Endocytosis Based on Megalin-Cubilin

Under extracellular physiological pH in the small intestine, IgG antibodies bind not to FcRn but to the megalin-cubilin complex. Interestingly, IgG was transported across the epithelium based on megalin-cubilin complex-mediated endocytosis followed by FcRn-mediated exocytosis through handoff in suckling rats [45]. Therefore, mAbs released from pH-sensitive nanoparticles at physiological pH in the small intestine can be transcytosed through a handoff. Nevertheless, it is true that FcRn and cubilin are present in the small intestine; however, whether megalin is expressed is controversial. It has been reported that megalin may be absent in the small intestine [48] (Table 2). Megalin is involved in inducing endocytosis rather than cubilin in the megalin-cubilin complex. It is unclear whether cubilin is expressed alone in the absence of megalin. Nevertheless, if it were not for megalin in the small intestine, there would be countermeasures. The light chain of IgG binds to megalin and cubilin [47,52]. Ligand-receptor clustering on the cell surface induces endocytosis [53,54]. Cubilin lacks a transmembrane part and is anchored to the apical membrane through its *N*-terminal. Amnionless is a transmembrane protein that forms a complex with cubilin. Structurally, an IgG molecule can bind to two cubilin molecules. Thus, clustering IgG-cubilin could induce endocytosis and dissociate in endosomes through acidification. Another approach is to preliminarily use a mixture of IgG and megalin, even though megalin is a single transmembrane protein with a cytoplasmic C-terminal. With or without megalin, IgG can be delivered across the small intestinal epithelium through translational substance exchange from megalin-cublin to FcRn in endosomes.

#### 2.7.2. Oral mAb Delivery Using Nanoparticles through Endocytosis Based on FcRn

The stomach is protected from its own gastric acid through two mucus layers. This suggests that H^+^ does not penetrate the mucus layer. The small intestinal epithelium is covered with a single mucus layer, which is different from the endothelium. H^+^ is poured into the lumen by the Na^+^/H^+^ exchangers in enterocytes. As a result, H^+^ accumulates between the mucus layer and cell surface [55]. Thus, mAbs released from the nanoparticles could bind to FcRn owing to locally acidic circumstances, following which they could be internalized through receptor-mediated endocytosis and liberated into the lamina propria after membrane fusion.

#### 2.7.3. Oral mAb Delivery Using Nanoparticles through Pinocytosis Based on FcRn

Pinocytosis is an energy-requiring bulk transport in constitutive processes or strictly controlled processes, and it non-selectively envelops fluids and certain molecules such as nutrients into vesicles. Accordingly, through pinocytosis bystander mAbs, which are released from the nanoparticles, are absorbed into the endosomes. The mAbs bind to FcRn in the endosomes through acidification, and they are released to the lamina propria. The mechanisms of pinocytosis are classified into several pathways, including macropinocytosis, clathrin-dependent endocytosis, caveolin-dependent endocytosis, and clathrin- and caveolin-independent endocytosis. It is difficult to induce systematic pinocytosis. Thus, a strategy that predominantly depends on pinocytosis is not efficient.

#### 2.7.4. Oral mAb Delivery Using Nanoparticles through Transcytosis in M Cells

M cells in Peyer’s patches, where the mucus layer is thin, absorb materials such as antigens through endocytosis. In fact, non-enteric NPs are endocytosed by M cells. However, it is unknown whether human M cells express FcRn. Moreover, the population of M cells is considerably smaller than that of enterocytes in the small intestine. Thus, a strategy based on transportation by M cells is not effective for mAb delivery through endocytosis using enteric pH-sensitive nanoparticles. Nonetheless, M cells would endocytose mAbs through mechanisms other than FcRn transcytosis. Eventually, mAb delivery based on transcytosis by enterocytes is more effective than that by M cells.

#### 2.7.5. Bio-Betters and Bio-Superiors

Repurposing existing mAb drugs approved for intravenous or subcutaneous administration for oral use may have limitations with respect to their effectiveness against the corresponding diseases. At present, bio-betters and bio-superiors based on protein engineering have been developed for use in biomedicines including Ab drugs [56]. Tuning affinity to FcRn might optimize oral mAb distribution. Accordingly, bispecific mAbs against target molecules such as cargos and membrane proteins as ligands on the surface of enterocytes could be transcytosed across the epithelium. Recycling Abs, including satralizumab, are engineered Abs that liberate their binding antigens in endosomes through acidification. Such free Abs are recycled back to the plasma membrane by FcRn, while the liberated antigens are subject to degradation in lysosomes. Recycling Abs exhibit a longer biological half-life than normal Abs. Sweeping Abs are functionalized recycling Abs that can bind reversibly to FcRn even under neutral pH, continue to bind to FcRn in endosomes, and liberate their antigen in endosomes through acidification; eventually, they are released from FcRn after endosomal fusion to the plasma membrane. Sweeping Abs can bind to FcRn at the extracellular physiological pH in the small intestine. Another approach is designing mAbs with mucoadhesive and mucopenetrating functionalities in order to enhance their transepithelial transport, even though enteric nanoparticles release their cargos in the lumen or the mucus layer, particularly at the jejunum, where peristaltic action is dynamically intense. Mucopenetrating functionality of Abs might be created by introducing the feature to avoid excessive electrostatic interaction with negative charge in mucins, although such electrostatic interaction is effective in mucoadhesion and transient adhesion to heparan sulfate proteoglycans on the surface of the epithelial cells. Furthermore, introducing modifications, such as resistance to gastric and intestinal proteases, can also be effective for improving bioavailability. Recently, nanobodies (15–45 kDa) known as single-domain Abs seem potential alternatives to Abs. However, most of them lack Fc domains. Thus, nanobodies are unlikely to be delivered across the intestinal epithelium to the systemic circulation based on FcRn-mediated transcytosis. Even though nanobodies are endocytosed based on pinocytosis, they would be degraded through the lysosomal degradative pathway. Nonetheless, nanobody-Fc domain conjugates could be transcytosed through the secretory pathway. The use of ingenuous strategies can enable the development of mAbs as promising pharmaceutical agents.

## 3. Conclusions

A wide range of drug modalities is being investigated for drug discovery and development. mAb drugs are used for molecular-targeted therapy, owing to their specific ability to bind to the corresponding antigens and thereby produce effective activity with minimal off-target side effects. However, clinically approved mAb drugs are administered through intravenous or subcutaneous routes, because mAbs are subjected to proteolysis and denaturation by proteases and gastric acid. Intravenous administration has disadvantages, such as pain, discomfort, stress, and potential infection. Thus, if oral mAb administration is established, patients will be free from such disadvantages. Accordingly, clinical trials have been performed for evaluating oral mAb administration in local gastrointestinal diseases. However, orally administered mAbs have not been approved for local use. Intriguingly, oral Abs in milk are transported by FcRn to the systemic circulation in neonates, probably because milk feeding neutralizes the gastric pH to 4–5 [10]. Furthermore, very small amounts (1–5%) of intact Abs have been detected in the systemic circulation, even through the oral administration route in adults [10]. Therefore, preventing mAbs from proteolytic digestion and denaturation by pepsin and gastric acid using an enteric nanodelivery system might enable mAb delivery across the intestinal epithelium to the systemic circulation. Released intact mAbs from enteric nanoparticles can be transferred to the vascularized lamina propria of the small intestine through RMT based on FcRn or megalin-cubilin, and they can subsequently enter the portal vein. IgG antibodies have a high affinity to FcRn under weakly acidic conditions and low or no affinity under physiological pH. Developing enteric nanoparticles is one of the most important factors for success, with regard to pH sensitivity in the small intestine, appropriate mucoadhesion as an anchor, and cargo encapsulation ability. Enteric Eudragit^®^ polymer is a promising candidate. In addition, SMART-Ig^®^, such as recycling Abs and, particularly, sweeping Abs that are modified to bind FcRn under neutral pH, can be protein engineering technologies that establish oral mAb delivery across the intestinal epithelial cells through FcRn-mediated transcytosis to the systemic circulation. From the standpoint of structuralism by Lévi-Strauss, structures such as social environments and rules unconsciously regulate human activities. Similarly, pharmacokinetics and pharmacodynamics are regulated by structures in living systems, including not only cells and tissues but also organic activity. In contrast, from the standpoint of existentialism by Sartre, existence precedes essence. By taking advantage of the existing machinery system in the living body using well-designed molecules such as functionalized nanoparticles, a new orderly system can be established. Thus, sophisticated medicinal chemists and pharmaceutical scientists must work with a sense of mission. Consequently, the development of innovative drugs will ease patient suffering in intractable diseases.

## Figures and Tables

**Table 1:** Summary of clinical trials focusing on orally administered Ab delivery described in this review.

#	Administrated Drug	Formulation/Co-Administrated Drug	Disease	Sponsor	Phase	Study Start Date	Study Completion Date	ClinicalTrials.gov Identifier (accessed on 23 January 2021)	Status	References
(i)	Oral V565		Crohn’s disease	VHsquared Ltd.	Phase 2	December 2016	December 2018	NCT02976129	Unknown	-
(ii)	Oral V565	Capsule	Ulcerative colitis	VHsquared Ltd.	Phase 1	October 2017	October 2017	NCT03705117	Completed	-
(iii)	Oral AVX-470	Enteric-coated capsule	Ulcerative colitis	Avaxia Biologics, Incorporated	Phase 1	February 2013	December 2013	NCT01759056	Completed	[23]
(iv)	Oral muromonab-CD3	Omeprazole	Ulcerative colitis	Brigham and Women’s Hospital	Phase 1 Phase 2	April 2011	May 2013	NCT01287195	Completed	[24]
(v)	Oral foralumab	Omeprazole	NASH, NAFLD, Type 2 diabetes mellitus	Tiziana Life Sciences, PLC	Phase 2	December 2017	June 2019	NCT03291249	Withdrawn	[25]
(vi)	Oral muromonab-CD3		NASH	Hadassah Medical Organization	Phase 2	September 2010	April 2011	NCT01205087	Completed	[26]
(vii)	Oral anti-CD3 mAb	Omeprazole	Chronic hepatitis C	Inspira Medical AB	Phase 2	November 2011	October 2013	NCT01459419	Unknown	-
(viii)	Oral omalizumab		Milk allergy	Hugh A Sampson, MD	Phase 2	August 2010	October 2015	NCT01157117	Completed	[27]
(ix)	Oral omalizumab		Peanut Allergy	Lynda Schneider	Phase 1Phase 2	February 2011	September 2013	NCT01290913	Completed	[28]
(x)	Subcutaneous omalizumab		Peanut Allergy, Multi-food Allergy	National Institute of Allergy and Infectious Diseases	Phase 3	July 2019	December 2023	NCT03881696	Recruiting	-
(xi)	Oral AGY	Capsule	Celiac disease	Igy Inc.	Phase 1	May 2014	August 2015	NCT01765647	Completed	-
(xii)	Oral AGY	Capsule	Celiac disease	Igy Inc.	Phase 2	October 2019	December 2022	NCT03707730	Recruiting	-
(xiii)	Oral Oralgam		Autistic Disorder, Gastrointestinal Diseases	PediaMed Pharmaceuticals	Phase 2	April 2005	June 2006	NCT00110708	Unknown	[29]
(xiv)	Oral TAO1	Tablet	Common Cold	Theranor s.p.r.l	Phase 1Phase 2	September 2012	August 2013	NCT01651715	Completed	[30]
(xv)	Oral anti-influenza antibody	Tablet	Influenza	Hadassah Medical Organization	Phase 1	January 2010	January 2011	NCT01026350	Unknown	-
(xvi)	Oral anti-CsbD bovine IgG, Oral anti-CS17 bovine IgG		Diarrhea	Johns Hopkins Bloomberg School of Public Health	Phase 2	January 2007	October 2007	NCT00524004	Completed	[31]
(xvii)	Oral anti-CFA/I bovine IgG, Oral anti-CfaE bovine IgG		Diarrhea	Johns Hopkins Bloomberg School of Public Health	Phase 1	March 2006	October 2006	NCT00435526	Completed	[32]
(xviii)	Oral SBI		Mucositis	MercyOne Des Moines Medical Center	Phase 2	January 2020	December 2021	NCT04239261	Recruiting	-
(xix)	Oral SBI		HIV-associated enteropathy	Entera Health, Inc	Not applicable	April 2013	September 2014	NCT01828593	Completed	[33]
(xx)	Oral SBI		Female reproductive cancer	Mayo Clinic	Phase 2	October 2013	October 2021	NCT01867606	Active, not recruiting	-
(xxi)	Oral SBI		Advanced COPD with cachexia	Medical University of South Carolina	Not Applicable	December 2013	April 2016	NCT02067377	Completed	-
(xxii)	Oral SBI		Diarrhea-predominant irritable bowel syndrome	Mayo Clinic	Not Applicable	June 2014	November 2016	NCT02163213	Completed	-
(xxiii)	Oral SBI		Irritable bowel syndrome	Louisiana State University Health Sciences Center in New Orleans	Not Applicable	March 2017	March 2017	NCT02609529	Completed	-
(xxiv)	Oral antibodies in colostrum		*Clostridium difficile*-associated diarrhea	Hadassah Medical Organization	Phase 2Phase 3	September 2011	November 2013	NCT00747071	Withdrawn	-
(xxv)	Oral mAbs	Enteric nanoparticles	Systemic treatment						Under analysis in Tashima lab	-

**Table 2:** Overview of protein and RNA expression of IgG-binding proteins.

#	Protein (Gene Name)	Protein Expression	Level	RNA Expression	NX Values
(i)	FcRn (FCGRT)	Cerebellum	Medium	Granulocytes	164.3
		Cerebral cortex	Low	Small intestine	125.2
		Hippocampus	Low	Colon	104.2
		Caudate	Low	Monocytes	100.2
		Lung	Low	Liver	87.4
		Testis	Low	Total PBMC	72.1
		Heart muscle	Low	Dendritic cells	70.7
(ii)	Megalin/LRP2 (LRP2)	Kidney	High	Kidney	61.7
		Parathyroid gland	Medium	Parathyroid gland	61.4
		Testis	Medium	Placenta	10.5
		Placenta	Low	Small intestine	0.4
		Small intestine	Not detected		
(iii)	Cubilin (CUBN)	Kidney	High	Kidney	79.7
		Small intestine	Low	Small intestine	28.9
				Epididymis	11.5
(iv)	FcγRI (FCGR1A, CD64A)	not shown		Monocytes	35.6
				Epididymis	33.2
				Granulocytes	25.5
				Small intestine	0.1
(v)	FcγRI (FCGR1B, CD64B)	not shown		Epididymis	36.9
				Granulocytes	21.8
				Monocytes	19.2
				Small intestine	0.4

RNA expression was based on consensus normalized expression (NX) data from the three transcriptomics, i.e., internally generated Human Protein Atlas (HPA) RNA-seq data, RNA-seq data from the Genotype-Tissue Expression (GTEx) project, and CAGE data from FANTOM5 project. PBMC stands for peripheral blood mononuclear cells.

## Data Availability

The human protein atlas can be found at https://www.proteinatlas.org/.
